# Screening of 31 genes involved in monogenic forms of obesity in 23 Pakistani probands with early-onset childhood obesity: a case report

**DOI:** 10.1186/s12881-019-0886-8

**Published:** 2019-09-05

**Authors:** Robina Khan Niazi, Anette Prior Gjesing, Mette Hollensted, Christian Theil Have, Dmitrii Borisevich, Niels Grarup, Oluf Pedersen, Asmat Ullah, Gulbin Shahid, Ifrah Shafqat, Asma Gul, Torben Hansen

**Affiliations:** 1 Department of Biological Sciences, International Islamic University, Islamabad, Pakistan; 2 Novo Nordisk Foundation Center for Basic Metabolic Research, Faculty of Health and Medical Sciences, University of Copenhagen, Copenhagen, Denmark; 3 Department of Biochemistry, Faculty of Biological Sciences, Quaid-i-Azam University, Islamabad, Pakistan; 4 Children Hospital, Pakistan Institute of Medical Sciences (PIMS), Islamabad, Pakistan; 5 Department of Molecular Biology, Shaheed Zulfiqar Ali Bhutto Medical University, PIMS, Islamabad, Pakistan

**Keywords:** Autosomal recessive, Bardet-Biedl syndrome 9, Compound heterozygous, Early-onset obesity, Monogenic obesity, Pakistani families, Consanguinity

## Abstract

**Background:**

Consanguine families display a high degree of homozygosity which increases the risk of family members suffering from autosomal recessive disorders. Thus, homozygous mutations in monogenic obesity genes may be a more frequent cause of childhood obesity in a consanguineous population.

**Methods:**

We identified 23 probands from 23 Pakistani families displaying autosomal recessive obesity. We have previously excluded mutations in *MC4R, LEP* and *LEPR* in all probands. Using a chip-based, target-region capture array, 31 genes involved in monogenic forms of obesity, were screened in all probands.

**Results:**

We identified 31 rare non-synonymous possibly pathogenic variants (28 missense and three nonsense) within the 31 selected genes. All variants were heterozygous, thus no homozygous pathogenic variants were found. Two of the rare heterozygous nonsense variants identified (p.R75X and p.R481X) were found in *BBS9* within one proband, suggesting that obesity is caused by compound heterozygosity. Sequencing of the parents supported the compound heterozygous nature of obesity as each parent was carrying one of the variants. Subsequent clinical investigation strongly indicated that the proband had Bardet-Biedl syndrome.

**Conclusions:**

Mutation screening in 31 genes among probands with severe early-onset obesity from Pakistani families did not reveal the presence of homozygous obesity causing variants. However, a compound heterozygote carrier of *BBS9* mutations was identified, indicating that compound heterozygosity must not be overlooked when investigating the genetic etiology of severe childhood obesity in populations with a high degree of consanguinity.

**Electronic supplementary material:**

The online version of this article (10.1186/s12881-019-0886-8) contains supplementary material, which is available to authorized users.

## Background

Worldwide, the prevalence of obesity has risen more than tenfold during the past four decades and approximately124 million children and adults, aged five to 19 years old, were obese in 2016 [1]. Obesity is one of the major risk factors for metabolic syndrome, including arterial hypertension, cardiovascular disease, diabetes mellitus, dyslipidemia and cancer [2, 3]. The etiology of obesity comprises both environmental and genetic factors, with a heritability of body mass index (BMI) between 40 to 70% [4, 5].

In rare monogenic forms of obesity, disruption of a single gene is the cause obesity and individuals typically display severe early-onset obesity along with hyperphagia and endocrine disorders [6, 7]. Most of the causative proteins in monogenic forms of obesity are acting in the hypothalamic leptin-melanocortin signalling pathway, which is essential for the regulation of food intake, body weight and energy regulation [8, 9].

Generally, mutations in *leptin* (*LEP*), the *leptin receptor* (*LEPR*) and the *melanocortin 4 receptor* (*MC4R*) represent the most common cause of monogenic forms of obesity and mutations within these genes have been demonstrated to cause childhood morbid obesity in probands of various ethnicities [10–14]. Yet, less than 5% of cases are explained by variants in these genes in out-bred populations [15] and possibly upto 30% in a consanguineous Pakistani population [12, 16].

Other genes are involved in the melanocortin signalling pathway and many of these have also been implicated in monogenic forms of obesity, including *POMC, PCSK*, *SIM1*, *BDNF*, *NTRK2*, *SH2B1* and *MRAP* [6].

The most distinct monogenic syndromic forms of obesity are characterized by severe early-onset obesity combined with other features, including alterations in hormone levels or dysmorphic characteristics, such as organ developmental deformities [17, 18]. Although a few syndromic forms of obesity, such as Alström syndrome, are not characterized by developmental delay [19], several are linked with varying degrees of mental retardation, including Prader-Willi syndrome [19], SIM1 syndrome [20] and WAGR syndrome [21]. Moreover, Bardet-Biedl syndrome (BBS), fragile X syndrome, Cohen syndrome and Albright’s Hereditary Osteodystrophy are all pleotropic disorders linked to developmental delay [19]. These rare syndromic forms of obesity may be instigated by either autosomal, X-linked chromosomal abnormalities or distinct genetic defects [18, 22, 23].

Worldwide, consanguineous marriage have been practiced in many populations for several generations due to social and economic benefits [24–26] and the Pakistani population has the highest rate of consanguinity in the world, with frequencies of 60–76% [27, 28]. In families with a known history of consanguineous marriages, the degree of homozygosity in family members is 11% on average and consanguinity thereby increases the risk of family members suffering from autosomal deleterious recessive disorders [28]. Thus genetic screening of consanguineous families with severe early-onset obesity, constitutes a powerful method of identifying causal homozygous mutations and has enabled the identification of rare damaging variants in e.g. *LEP*, *LEPR* and *MC4R* [10, 11, 29].

In the current study, we performed genetic screening of 31 genes previously demonstrated to be involved in childhood obesity in 23 unrelated probands from Pakistani families with severe early-onset obesity segregating as an autosomal recessive trait. We have previously excluded mutations in *LEP*, *LEPR* and *MC4R* as causative mutations in all probands.

## Methods

### Study population

**Fig. 1:**
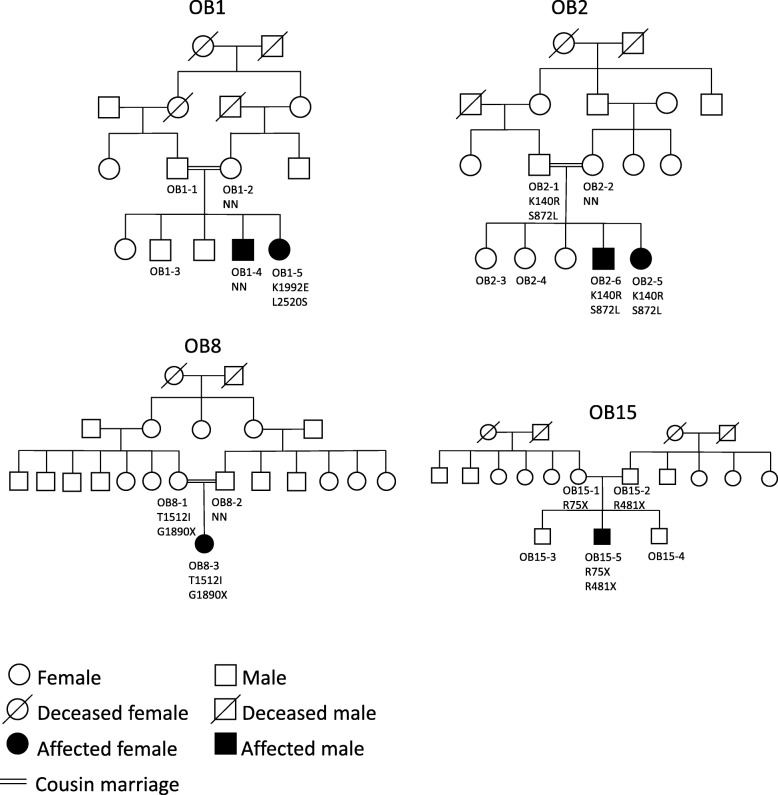
Pedigrees of families in which probands carry two mutations within the same gene. Possibly causal variant are denoted under the carrier ID

Twenty-three families originating from different regions of Pakistan were recruited for the current study. These families were examined at Children Hospital, Pakistan Institute of Medical Sciences (PIMS), Islamabad. Patients were recruited between November 2015 and April 2017. Eleven families had known consanguineous marriages. Selection of families was based on four parameters, including: 1) BMI of probands > 30 kg/m^2^ or BMI standard deviation score (SDS) ≥3; 2) Probands displaying obesity onset before five years of age; 3) Parents of the probands having a BMI < 25 kg/m^2^, consistent with an autosomal recessive mode of inheritance and 4) Probands not carrying homozygous *LEP*, *LEPR* and *MC4R* mutations. In families with several affected individuals, the patient having the most severe phenotype was selected for sequencing (Table 1). Probands from all 23 families in addition to family members from OB1 (OB1–2 and OB1–4), OB2 (OB2–1, OB2–2 and OB2–6), OB8 (OB8–1 and OB8–2) and OB15 (OB15–1 and OB15–2) underwent targeted resequencing (Fig. 1).

**Table 1:** Clinical presentation of probands

Trait	Probands
Gender (M/F)	13/10
Age at enrolment (years)	16.6 (6.81)
Age of obesity onset (years)	Below the age of 5 years
Height (cm)	149.8 (24.4)
Weight (kg)	82.8 (26.0)
BMI (kg/m^2^)	36.5 (8.81)
BMI SDS	3.20 (1.24)
Waist circumference (cm)	102.0 (14.7)
Consanguine family (yes/no)	11/12
Family history of obesity (yes/no)	14/9

Data is presented as neither number of individuals or as mean (SD)

### Clinical characterization

Height (cm) and waist circumference (cm) were measured with non-extendable plastic tape with the participant standing in a straight position and without shoes. Using a digital scale, weight (kg) was measured to the nearest 1.0 kg with the participant wearing light clothes and no shoes. Based on interviews, information about the age at obesity onset, other major chronic disease or metabolic diseases (if any) segregating in the family, eating habits and physical activity were collected along with obesity-related co-morbidities. BMI was calculated as the weight in kilograms divided by the square of the height in meters (kg/m^2^) and a BMI standard deviation score (SDS) was calculated based on a World Health Organization (WHO) reference population [30] using the LMS method [31]. The clinical characteristics of the study participants are presented in Table 1 and Additional file 1.

For comparison of inbreeding coefficient, we used information from 298 Danish individuals from 61 families which have previously been described [32].

### Genomic DNA extraction

Approximately 3–5 ml venous blood samples from 31 affected and 79 unaffected family members were collected in the non-fasting state in 8.5 ml vacutainer tubes (BD Vacutainer® ACD, Franklin Lakes NJ, USA). Following the standard phenol-chloroform method [33] and using QIAamp DNA Mini Kit (Qiagen, Germany), the genomic DNA was extracted.

### Targeted region array design

A chip-based customized nucleotide probe was used for capturing genomic DNA of the coding regions of 31 selected genes involved in monogenic forms of obesity (*ADCY3, ALMS1, ARL6, BBS1, BBS2, BBS4, BBS5, BBS7, BBS9, BBS10, BBS12, BDNF, CCDC28B,CEP290, CREBBP, EP300, GNAS, IER3IP1, MKKS, MKS1, MRAP2, NTRK2, PCSK1, PHF6, POMC, SH2B1, SIM1, TMEM67, TRIM32, TTC8* and *VPS13B*). Methods for DNA extraction, target region capture and next generation sequencing have previously been described [34].

The final captured DNA libraries were sequenced according to the manufacturer’s standard cluster generation and sequencing protocols, using the Illumina HiSeq2000 Analyzers as paired-end 90 base pair reads.

### Genotyping

Illumina Infinium Human CoreExomeBeadChip (CoreExomeChip) genotyping was performed in 104 individuals from 23 families of Pakistani origin and 298 individuals from 61 families of Danish origin using Illumina’s HiScan system at the laboratory facilities of the Novo Nordisk Foundation Center for Basic Metabolic Research at Symbion, Copenhagen, Denmark. The pipeline and quality control protocol has previously been described for Pakistani participants [16] and for Danish participants [32].

### Variant selection

Variants were considered possibly pathogenic if: 1) they were variants with minor allele frequency (MAF) < 0.1% in publically available databases [35, 36]; 2) coding non-synonymous variants or splice variants located up to 3 nucleotides into the intron/exon boundary; 3) having minimum depths of 20x and 4) a allelic ratio between 0.4–0.6 for heterozygous mutations.

Families were recruited based on the presence of affected children from non-affected parents both from families with known and without known consanguinity. Thus, three different genetic inheritance patterns may likely exist in the recruited families: 1) recessive, 2) compound heterozygotes and 3) heterozygous de novo heritance. The latter mode of inheritance was not considered despite the fact that causal heterozygous mutations have been suggested for some of the selected genes [37–40], as authentication of such potentially causal heterozygous de novo mutations is complex and sequencing information from both parents is warranted.

Pathogenicity of the variants were evaluated using in silico annotation tools [41–43] especially using Combined Annotation Dependent Depletion (CADD) score where a PHRED-scaled CADD score above 10 predicts pathogenicity in top 10 percentile of all variants and a score above 20 predicts the top 1 percentile [44].

### Statistical analysis

Relatedness analysis was calculated as an inbreeding coefficient which is based on the genotyping of included individuals. This estimates the probability of a random locus in related individuals being identical by descent. This was calculated using the “het” command using PLINK [45]. Inbreeding coefficients was compared between individuals from consanguine Pakistani families, non-consanguine Pakistani families and Danish out-breed families using a student’s t-test in R software (version 3.2.3; R Foundation for Statistical Computing, Boston, MA, USA).

## Results

We investigated the level of relatedness in families with and without known consanguinity using the inbreeding coefficient. This estimates the probability of a random locus in related individuals being identical by descent. We found that families with known consanguinity had a mean inbreeding coefficient of 5.6% (SD: 4.5) in contrast to Pakistani families without consanguinity having a mean inbreeding coefficient of 3.2% (SD: 3.0) (*p* = 0.003). However, when comparing non-consanguine Pakistani families to Danish outbred families having an inbreeding coefficient of − 1.02% (SD: 0.65), the inbreeding coefficient of non-consanguine Pakistani families was still significantly higher (*p* = 4*10^− 13^). Thus, recessive inheritance-patterns are likely to exist in Pakistani families with both known and unknown consanguinity.

Thirty-one genes were selected based on their known causal involvement in childhood obesity and among the probands we identified a total of 31variants located in *ALMS1*, *BBS7*, *BBS9*, *BBS10*, *CREBPB*, *EP300*, *PCSK1*, *POMC* and *VSP13B* fulfilling the criteria for being possibly pathogenic (28 missense and three nonsense) (Additional file 2).

Due to the large number of consanguineous families and the high level of relatedness in families without known consanguinity, we searched for homozygous recessive variants. Yet, no homozygous pathogenic carriers were found.

Subsequently we investigated the presence of probands carrying two heterozygous mutations within the same gene and found four potentially compound heterozygous probands: 1) OB1–5 carrying the p.K1992E and the p.I2520S in *ALMS1*; 2) OB2–5 carrying the p.S872 L and p.K140R in *CEP290*; 3) OB8–3 carrying the p.T1512I and p.G1890X also in *CEP290*; and 4) OB15–5 carrying the p.R75X and p.R481X in *BBS9* (Table 2).

**Table 2:** Mutation type and phenotypic presentation in probands carrying two heterozygous variants within the same gene

Family ID	Proband ID	Gene	Identified variants	Primary phenotype of patient	Secondary phenotypes of patients	Phenotypic characteristic of patients with syndromes related to investigated gene	Mutations co-segregation with phenotype
OB1	OB1.5	*ALMS1*	p.K1992E p.L2520S	Hyperphagia	NA	ALMS: retinal degeneration, hearing loss, diabetes mellitus, dilated cardiomyopathy, urological dysfunction, pulmonary, hepatic, renal failure	No
OB2	OB2–5	*CEP290*	p.S872 L p.K140R	Hyperphagia, Hypertension	NA	Joubert syndrome: brain abnormalities, molar tooth sign, hypotonia, ataxia, hyperpneaorapnea, ocular motor apraxia	No
OB8	OB8–3		p.T1512I p.G1890X	Dyslipidemia	NA		No
OB15	OB15–5	*BBS9*	p.R75X p.R481X	Hypogonadism, Mental retardation, Obesity, Vision impairment.	Speech impairment, Hypertension.	Bardet-bieldel syndrome: obesity, polydactyly, renal anomalies, retinopathy, mental retardation	Yes

OB1–5, carrying two rare missense variants in *ALMS1*, displays hyperphagia in addition to severe early-onset obesity. Sequencing of the mother (OB1–2) and the affected sibling OB1–4) revealed that the variants found in the proband (OB1–5) was not carried by the affected sibling, nor was the mother a carrier of any of the two *ALMS1* variants found in proband (Fig. 1). In addition, the affected individuals in OB1 did not present with the phenotypic characteristics of Alstrom syndrome such as retinal degeneration, hearing loss, diabetes mellitus, dilated cardiomyopathy (DCM), urological dysfunction, pulmonary, hepatic and renal failure. Thus, neither co-segregation nor phenotypic presentation suggests that the two variants found in OB1–5 in *ALMS1* are causal for the childhood obesity in OB1.

The probands in family OB2 and OB8 are each having two mutations in *CEP290*. OB2–5 carried the p.K140R and p.S872 L missense mutations and the proband in OB8 (OB8–3) is carrying the p.T1512I missense mutation and the p.G1890X nonsense mutation. The functional prediction of the p.K140R variant based on the CADD score, indicate only a minor impaired functionality (CADD score:14.6, Table 2). This lack of presumed functionality is supported by the lack of clinical characteristic in OB2 of patients with *CEP290* mutations such as retinal degeneration, hypogonadism, polydactyly, renal dysfunction and MR [46]. Subsequent sequencing of *CEP290* in the parents (OB2–1 and OB2–2) and sibling (OB2–6) of OB2–5, revealed that both variants present in the proband were inherited from the father but not from the mother in whom none of the two variants were present (Table 2).

The p.G1890X variant found in OB8–3 has previously been found to cause Joubert syndrome-related disorders (JBTS) in a homozygous manner in a Turkish family [47]. The JBTS affects the central nervous system (brain and spinal cord), retina and kidney and it is inherited in autosomal recessive manner. Moreover, a high CADD scores was found for both the missense and nonsense variants (26.5 and 36, respectively) supporting a highly pathogenic nature of these two mutations. Yet, sequencing of *CEP290* in the parents (OB8–1 and OB8–2) of proband OB8–3 showed that the variants found in OB8–3, both were inherited from the mother (Fig. 1). This lack of co-segregation was also supported by the proband (OB8–3) not displaying any of the symptoms characteristic of the syndromes related to *CEP290* mutations such as JBTS, thus, we do not believe OB8–3 is suffering from *CEP290* related obesity.

The probands in OB15 is carrying the two nonsense mutations p.R75X and p.R481X both very likely highly deleterious mutations. Patients with BBS caused by homozygous or compound heterozygous mutations in *BBS9* are characterized by obesity, polydactyly, renal anomalies, retinopathy and mental retardation. OB15–5 presents with a large number of the primary BBS phenotypes including hypogonadism, developmental delay with learning difficulties, speech- and vision- impairment in addition to severe childhood obesity. Moreover, sequencing of *BBS9* in the parents (OB15–1 and OB15–2) of OB15–5 revealed that p.R75X was inherited from the mother and p.R481X was inherited from the father. Therefore, the proband OB15–5 is likely a patient with BBS due to compound heterozygous mutations in *BBS9*.

## Discussion

In the current study, targeted resequencing of the coding regions of 31 selected genes known to be involved in monogenic forms of obesity (excluding *LEP*, *LEPR* and *MC4R)* was performed in 23 probands from Pakistani families with severe early-onset obesity segregating as an autosomal recessive trait. One compound heterozygous proband was identified carrying two nonsense variants in *BBS9/PTHB1* (p.R75X and p.R481X) in exon 3 and exon 14, respectively, causing BBS.

Homozygous and tri-allelic variants in *BBS* genes have been reported to cause BBS phenotypes in Pakistani population [48–50], but no prior *BBS9* compound heterozygous patients have been reported in non-consanguineous Pakistani families.

Bardet-Biedl syndrome protein complex (BBSome) is a central entity of ciliogenesis and it has 10 subunits from BBS1 to 10 [51]. BBS9, a99-kDa protein, is one of the component of the BBSome and it has a suggested role in associating other subunits [52]. Studies of BBS9 function in knock down mouse and zebra fish, have revealed its significant role in cilia biogenesis [53]. In our study, the identified variant p.R75X is positioned in the N-terminal domain and the p.R481X is positioned in the C-terminal half of the PTHB1 protein. The full length BBS9 contains 887 amino acids, thus, termination of the protein after only 75 and 481 amino acids, respectively, is not surprisingly detrimental for the function of the protein due to non-sense mediated decay or production of truncated protein [54]. Hence, we believe that these loss-of-function mutations in *BBS9* may be responsible for structural abnormality in cilia due to reduced integrity of BBSome proteins complex.

Previous studies examining the genetic causes of severe early-onset obesity in Pakistani families have mainly focused on a few genes most often linked to monogenic forms of obesity i.e. *LEP*, *LEPR* and *MC4R* [10, 12, 55–57], yet, more recently, a mutation screen of multiple genes was performed in 39 unrelated children with severe obesity from consanguineous Pakistani families [56]. The study included 21 of the 31 genes examined in the present study and was similar to our findings with no casual mutations in homozygous conditions identified [56]. The remaining ten genes (*CCDC28B, CREBBP, EP300, IER3IP1, MRAP2, PHF6, SH2B1, TMEM67, VPS13B),* selected in the present study were therefore, screened for the first time in Pakistani families with the aim of assessing the prevalence of damaging mutations conferring early-onset obesity. However, our findings indicate that mutations within the selected 31 genes are not a common cause of severe early-onset obesity in the Pakistani population.

No personalized treatment approach has been identified for syndromic forms of obesity and the affected individuals are generally advised to follow current general treatment approaches, including increased physical activity, psychomotricity (activities which integrate cognitive, emotional and physical elements) and an energy restricted diet [6].

Four potential compound heterozygous probands were identified; however, we only claim the causal effect in one proband. Establishing pathogenicity of missense mutation is challenging and this even more so in the present Pakistani study population, as identified variants may be present in the unaffected background population but may not appear in any publically available databases which are most frequently based on Caucasian populations. However, in the present study, co-segregation analysis supported the likely causality of nonsense mutations in which pathogenicity is highly probable.

Our investigation of the relatedness between individuals in families with known consanguinity versus families without known consanguinity, clearly indicate that the fraction of genetic loci which show identity by descent within a family, are of considerable proportion even in families without known consanguinity from population where consanguinity is frequent. This strongly indicates that recessive disease mechanisms generally should be considered in families of Pakistani descent. Thus, whole exome sequencing or whole genome sequencing will likely be fruitful strategies to identify novel causal homozygous mutations in inbred populations.

## Conclusion

Among 23 Pakistani families, mutations within 31 genes known to be involved in the development of obesity are not a cause of severe early-onset obesity. Yet, the present study identified one compound heterozygote patient with BBS, thus, the presence of compound heterozygous patients must not be overlooked in populations with a high degree of consanguinity.

## Additional files

Additional file 1: Clinical information on 23 probands with early onset childhood obesity from 23 Pakistani families (DOCX 23 kb)

Additional file 2: List of identified rare variants among 23 Pakistani probands with early onset childhood obesity. (DOCX 26 kb)

## Data Availability

The datasets generated and/or analyzed during the current study are not publicly available due to limitations in the consent form (consent has been given to study severe early-onset obesity), but are available from the corresponding author on reasonable request.

## Abbreviations

BBS9: Bardet-Biedl syndrome 9
BMI: Body mass index
LEP: Leptin
LEPR: Leptin-receptor
MAF: Minor allele frequency
MC4R: Melanocortin 4 receptor
PIMS: Pakistan Institute of Medical Sciences
SDS: Standard deviation score
WES: Whole exome sequencing
