# Supplementary material for: Screening of 31 genes involved in monogenic forms of obesity in 23 Pakistani probands with early-onset childhood obesity: a case report
Source: BMC Med Genet. 2019 Sep 5;20:152. doi: 10.1186/s12881-019-0886-8 (PMC6727494; doi:10.1186/s12881-019-0886-8)
Supplement: Supplementary file 1 — Clinical information on 23 probands with early onset childhood obesity from 23 Pakistani families (DOCX 23 kb) [file 12881_2019_886_MOESM1_ESM.docx]

**Additional file 1: Clinical information on 23 probands with early onset childhood obesity from 23 Pakistani families**

| **Family ID** | **Consanguine family** | **Subject ID** | **Age range of enrolment (yrs)** | **Age of obesity onset (yrs)** | **Height (cm)** | **Weight**  **(kg)** | **BMI (kg/m^2^)** | **BMI SDS** | **Waist circumference (cm)** | **Family history of obesity** | **Obesity-related co-morbidities** | **Family-related disorders** |
| --- | --- | --- | --- | --- | --- | --- | --- | --- | --- | --- | --- | --- |
| OB1 | Yes | OB1-5 | 8-10 | < 5 | 122.0 | 98.0 | 66.0 | 4.78 | N/A | no | hyperphagia | hypertension |
| OB2 | Yes | OB2-5 | 22-24 | < 5 | 155.0 | 97.0 | 40.4 | 3.67 | 111.8 | no | hyperphagia, hypertension | hypertension |
| OB3 | Yes | OB3-6 | 8-10 | < 5 | 137.1 | 82.0 | 43.6 | 4.16 | 101.6 | yes | hyperphagia | none |
| OB5 | Yes | OB5-5 | 12-14 | < 3 | 137.0 | 75.0 | 34.0 | 3.63 | 91.4 | yes | hyperphagia, hypertension | diabetes,hypertension, asthma, heart disease |
| OB6 | No | OB6-5 | 16-18 | < 3 | 152.4 | 67.0 | 28.9 | 1.93 | 86.3 | yes | none | None |
| OB7 | No | OB7-3 | 14-16 | < 5 | 162.5 | 77.0 | 29.2 | 2.36 | 101.6 | yes | hypertension, gynaecomastia | diabetes, hypertension, heart disease, nephropathy |
| OB8 | Yes | OB8-3 | 6-8 | < 5 | 101.6 | 32.0 | 31.0 | 4.48 | 78.7 | yes | dyslipidaemia | diabetes, hypertension |
| OB9 | No | OB9-5 | 22-24 | < 5 | 177.8 | 132.0 | 41.8 | 3.11 | 111.8 | yes | hyperphagia, hypertension | diabetes, hypertension, asthma |
| OB10 | Yes | OB10-4 | 22-24 | ~ 5 | 162.5 | 81.0 | 30.7 | 2.10 | 111.8 | no | polycystic ovary, hypertension | CVD, hypertension |
| OB11 | No | OB11-5 | 16-18 | < 5 | 172.7 | 108.0 | 36.2 | 3.12 | 111.8 | yes | hyperphagia | diabetes, hypertension |
| OB12 | Yes | OB12-4 | 26-28 | ~ 5 | 162.5 | 104.0 | 39.3 | 2.32 | 106.6 | yes | gout, ulcer, epilepsy, hypertension | diabetes, hypertension, osteoporosis, arthritis |
| OB13 | No | OB13-6 | 12-14 | < 5 | 155.0 | 70.0 | 29.2 | 2.68 | 99.0 | yes | hypertension, nephropathy, chronic fatigue | asthma |
| OB14 | Yes | OB14-5 | 12-14 | < 5 | 157.5 | 80.3 | 32.4 | 2.78 | 109.2 | yes | hyperphagia | diabetes, hypertension |
| OB15 | No | OB15-5 | 16-18 | ~ 2 | 152.4 | 76.0 | 32.7 | 2.73 | 104.1 | no | developmental delay, CVD | none |
| OB16 | No | OB16-4 | 12-14 | ~ 2 | 162.5 | 66.0 | 25.0 | 2.02 | 86.3 | yes | hyperphagia | diabetes |
| OB17 | No | OB17-3 | 0-2 | after 3 months | 76.2 | 20.0 | N/A | 7.37 | 58.4 | no | hyperphagia | none |
| OB18 | No | OB18-3 | 22-24 | < 5 | 162.5 | 89.0 | 33.7 | 2.64 | 109.2 | yes | hypertension | diabetes, hypertension, heart disease, nephropathy |
| OB19 | No | OB19-4 | 14-16 | < 5 | 139.7 | 92.0 | 47.1 | 3.93 | 106.6 | no | hyperphagia | hypertension |
| OB20 | No | OB20-4 | 18-20 | ~ 5 | 162.5 | 82.0 | 31.0 | 2.26 | 104.1 | no | hyperphagia | none |
| OB21 | Yes | OB21-4 | 20-22 | < 5 | 142.2 | 85.0 | 42.0 | 3.66 | 114.3 | yes | heart problem, hyperphagia | none |
| OB22 | No | OB22-3 | 20-22 | ~ 5 | 177.8 | 122.0 | 38.6 | 3.22 | 119.3 | no | hypertension | none |
| OB23 | Yes | OB23-5 | 30-32 | ~ 5 | 177.8 | 115.0 | 36.4 | 1.57 | 124.4 | yes | hypertension, varicose vein problem | hypertension, diabetes, nephropathy, metabolic syndrome |
| OB24 | Yes | OB24-10 | 10-12 | 1.5 | 137.0 | 53.0 | 28.2 | 2.98 | 96.5 | no | hyperphagia, fatigue, continuous head movement, weak eye sight | nephropathy, asthma, CVD, arthritis |
