# Supplementary material for: Screening of 31 genes involved in monogenic forms of obesity in 23 Pakistani probands with early-onset childhood obesity: a case report
Source: BMC Med Genet. 2019 Sep 5;20:152. doi: 10.1186/s12881-019-0886-8 (PMC6727494; doi:10.1186/s12881-019-0886-8)
Supplement: Supplementary file 2 — List of identified rare variants among 23 Pakistani probands with early onset childhood obesity. (DOCX 26 kb) [file 12881_2019_886_MOESM2_ESM.docx]

**Additional file 2: List of identified rare variants among 23 Pakistani probands with early onset childhood obesity.**

| **Sr. No.** | **Variant** | **Type of mutation** | **rs Number** | **MAF (gnomAD)** | **CADD score** | **Number of proband carriers** |
| --- | --- | --- | --- | --- | --- | --- |
| **Gene (transcript)** | ***ALMS1 (*NM_015120)** | | | | | |
| 1 | p.T707A | Missense | rs571389435 | 0.0002607 | 9.91 | OB17.3 |
| 2 | p.T1088R | Missense | rs556855697 | 0.0003710 | 19.2 | OB3.6 |
| 3 | p.S1896L | Missense | rs757128530 | 0.000008140 | 22.4 | OB10.4 |
| 4 | **p.K1992E** | **Missense** | **.** | **0** | **24.3** | **OB1.5** |
| 5 | p.R2344Q | Missense | rs759366425 | 0.00002443 | 12.97 | OB6.5 |
| 6 | p.S2415G | Missense | . | 0.000008128 | 26.1 | OB21.4 |
| 7 | **p.L2520S** | **Missense** | **rs776090716** | **0.00001807** | **27.7** | **OB1.5** |
| 8 | p.E3929G | Missense | rs771370411 | 0.00002031 | 28.7 | OB9-5 |
| **Gene (transcript)** | ***BBS7* (NM_176824)** | | | | | |
| 9 | p.E348G | Missense | rs575431546 | 0.00008955 | 25.8 | OB3.6 |
| **Gene (transcript)** | ***BBS9* (NM_014451.3)** | | | | | |
| 10 | **p.R75X** | **Nonsense** | **rs775081992** | **0.00002438** | **37** | **OB15.5** |
| 11 | p.C270S | Missense | rs763742314 | 0.00004879 | 20.6 | OB18.3 |
| 12 | **p.R481X** | **Nonsense** | **rs748601675** | **0.000004075** | **40** | **OB15.5** |
| 13 | p.A672T | Missense | rs751173437 | 0.00001220 | 24 | OB9.5 |
| 14 | p.E713V | Missense | rs61764068 | 0.0007593 | 28 | OB10.4 |
| **Gene (transcript)** | ***BBS10 (*NM_024685)** | | | | | |
| 15 | p.R422Q | Missense | rs138961848 | 0.0003574 | 12.57 | OB9.5 |
| **Gene (transcript)** | ***CEP290*(NM_025114)** | | | | | |
| 16 | p.R20H | Missense | . | 0 | 23.4 | OB21.4 |
| 17 | **p.K140R** | **Missense** | **rs750776051** | **0.000008128** | **14.6** | **OB2.6**  **OB2.5** |
| 18 | **p.S872L** | **Missense** | **rs373341530** | **0.0002599** | **24.7** | **OB2.6**  **OB2.5** |
| 19 | p.E905D | Missense | . | 0 | 24.1 | OB18.3 |
| 20 | **p.T1512I** | **Missense** | **.** | **0** | **26.5** | **OB8.3** |
| 21 | **p.G1890X** | **Nonsense** | **rs137852832** | **0.00009589** | **36** | **OB8.3** |
| **Gene (transcript)** | ***CREBBP* (NM_001079846)** | | | | | |
| 22 | p.T879A | Missense | NA | 0 | 8.825 | OB12.4 |
| **Gene (transcript)** | ***EP300* (NM_001077489)** | | | | | |
| 23 | p.P79S | Missense | . | 0 | 21.6 | OB2.6 |
| 24 | p.R155C | Missense | rs755050269 | 0.00008312 | 21.2 | OB20.4 |
| 25 | p.N248S (only in NM_001429) | Missense | rs762095513 | 0.00004874 | 8.69 | OB17.3 |
| 26 | p.S268L | Missense | rs566198679 | 0.00001639 | 15.85 | OB24.10 |
| 27 | p.R321P | Missense | . | 0 | 35 | OB17.3 |
| **Gene (transcript)** | ***PCSK1* (NM_000439)** | | | | | |
| 28 | p.N127I | Missense | rs574780528 | 0.0002275 | 21.3 | OB2.6  OB2.5 |
| 29 | p.T366M | Missense | rs369762633 | 0.00004072 | 23 | OB5.5 |
| **Gene (transcript)** | ***POMC* (NM_000939)** | | | | | |
| 30 | p.E30D | Missense | rs758258712 | 0.00001219 | 24 | OB7.3 |
| **Gene (transcript)** | ***VPS13B* (NM_017890)** | | | | | |
| 31 | p.T962M | Missense | rs547184348 | 0.0002167 | 27.1 | OB13.6 |

Bold variants are considered to be possible compound heterozygous variants.
